# Frequency of nosocomial influenza cases in a tertiary hospital during the 2012-13 to 2014-15 seasons

**DOI:** 10.1186/2047-2994-4-S1-O59

**Published:** 2015-06-16

**Authors:** JL Mendoza García, JG Mora Muriel, I Tenza Iglesias, P García Shimizu, M Fuster Perez, V García Roman, JL Carretero Ares, M El Attabi, J Sánchez Payá

**Affiliations:** 1 Hospital General Universitario de Alicante, Alicante, Spain

## Introduction

Control of influenza in the hospital must be a priority within the programs to improve patient safety.

## Objectives

The objective is to determine the frequency and characteristics of nosocomial influenza cases in a tertiary hospital in the last three seasons.

## Methods

We included all the patients hospitalized with suspected influenza during the 2012-13 and 2013-14 complete seasons; for the 2014-15, patients were included until week 10-2015. Cases were patients with influenza-like illness who were microbiologically confirmed (nasopharyngeal swabs or PCR positive for influenza virus A/B). Study variables were: type of infection (nosocomial -the onset of symptoms was 72 hours or more after the admission of the patient- / community-acquired), type of case (serious / not serious), death (yes / no). To study the association between seasons, chi-square test was used.

## Results

Among the confirmed influenza cases, the frequency of nosocomial cases was 14.2% (18/127) in the 2012-13 season, 11.6% (26/224) in the 2013-14 season and 14.1% (32/226) in the 2014-15 season; p = 0.672. The frequency of nosocomial cases among severe cases was: 7.5% (3/40) in the 2012-13 season, 6.1% (4/66) in the 2013-14 season and 8.9 (4/45) in the 2014-15 season; p = 0.904. The frequency of exitus in nosocomial cases was: 40% (2/5) in the 2012-13 season, 0% (0/11) in the 2013-14 season and 8.3% (1/12) in the 2014-15 season; p = 0.05.

## Conclusion

This study highlights the impact of nosocomial transmission of influenza within a tertiary hospital. The annual influenza vaccination of health professionals, together with recommendations on respiratory hygiene and an appropriate degree of compliance with droplet recommendations in the flu cases are the main strategies for the prevention of nosocomial influenza and therefore for the improvement of patient safety.

## Disclosure of interest

None declared.

